# Long‐term safety and efficacy of left atrial appendage closure in patients with small appendage orifices measured with transesophageal echocardiography

**DOI:** 10.1002/clc.23950

**Published:** 2022-11-15

**Authors:** Binhao Wang, Zhao Wang, Huimin Chu, Bin He, Guohua Fu, Mingjun Feng, Xianfeng Du, Jing Liu, Yibo Yu

**Affiliations:** ^1^ Arrhythmia Center Ningbo First Hospital Ningbo China; ^2^ Key Laboratory of Precision Medicine for Atherosclerotic Diseases of Zhejiang Province Ningbo China; ^3^ Department of Ultrasonography Ningbo First Hospital Ningbo China

**Keywords:** atrial fibrillation, left atrial appendage closure, stroke, Watchman

## Abstract

**Background:**

The Watchman device is the most widely used occluder but is indicated in atrial fibrillation (AF) patients with a maximal left atrial appendage (LAA) orifice diameter between 17 and 31 mm. We aimed to compare the long‐term safety and efficacy of left atrial appendage closure (LAAC) between patients with a small LAA (<17 mm) and those with an indicated LAA (17–31 mm) measured by transesophageal echocardiography (TEE).

**Methods:**

A total of 369 AF patients treated with LAAC between March 2015 and February 2019 were included and divided into two groups based on the maximal LAA orifice diameter measured by TEE: small LAA group (*n* = 22) and indicated LAA group (*n* = 347). Periprocedural complications and long‐term clinical outcomes were compared.

**Results:**

The Watchman device was successfully implanted in all patients. Mean device compression was higher in the small LAA group. Four patients (1.2%) in the indicated LAA group experienced pericardial effusion, and none experienced pericardial effusion in the small LAA group. Device‐related thrombus was detected in one (4.5%) patient in the small LAA group and five (1.4%) in the indicated LAA group (*p* = .310). After a mean follow‐up period of 4.1 ± 1.6 years, one patient in the small LAA group (4.5%; 1.1/100 person‐years) and four in the indicated LAA group (1.2%; 0.3/100 person‐years) suffered an ischemic stroke (*p* = .266).

**Conclusions:**

The safety and efficacy of LAAC with the Watchman device were comparable between patients with small and indicated LAA orifice diameters measured by TEE.

## 1 INTRODUCTION

Atrial fibrillation (AF) is the most sustained arrhythmia in the clinical setting and increases the risk of thromboembolism (TE).^1^ The majority of the thrombi originate in the left atrial appendage (LAA).^2^ Percutaneous left atrial appendage closure (LAAC) has now been considered an optional alternative in patients with contraindications to long‐term oral anticoagulation (OAC).^3^, ^4^ The Watchman device (Boston Scientific) is now the most widely used occluder but is indicated in patients with a maximal LAA orifice diameter between 17 and 31 mm.^5^ However, the LAA anatomy is highly heterogeneous, with a range of ostia diameters from 5 to 40 mm.^6^ An off‐label attempt is necessary to widen the indication of the Watchman device in a complex LAA anatomy to leave no man behind. Deploying a round occluder into a relatively small LAA may lead to an excessive compression rate, which could theoretically result in endocardium perforation or device embolization.^7^ However, overcompression was not uncommon in clinical practice.^8^ Therefore, overcompression to some extent maybe not be a hazard.

Few studies have reported LAAC in patients with small LAA orifices who have a high risk of TE.^9^, ^10^ Recently, Venkataraman et al.^9^ reported that LAAC with the Watchman device can be successfully and safely achieved in patients with a maximal LAA orifice diameter <17 mm. However, the study was a one‐arm observational investigation with short‐term follow‐up. Transesophageal echocardiography (TEE) is the current standard to assess LAA size in patients considered for LAAC with the Watchman device. Therefore, the present study aimed to compare the long‐term safety and efficacy of LAAC between patients with a small LAA orifice (<17 mm) and those with the indicated LAA orifice diameter (17–31 mm) measured by TEE.

## 2 METHODS

### 2.1 Study population

A total of 369 patients with nonvalvular AF undergoing percutaneous LAAC with the Watchman device at our center from January 2015 to December 2020 were retrospectively included. All clinical database and blood test results, echocardiography, and periprocedural baseline data were collected. Patients were excluded from the study if they had a thrombus in the LAA or left atrium (LA) detected by TEE, AF in the setting of moderate‐to‐severe mitral stenosis and/or in the presence of a mechanical heart valve, acute myocardial infarction or unstable angina, prior stroke or transient attack (TIA) within 30 days, uncontrolled hemorrhagic disease, or the presence of an atrial septal repair or a closure history. This study was conducted in compliance with the law protecting personal data in accordance with the guidelines of the Helsinki Declaration. The study was approved by the Ethics Committee of Ningbo First Hospital, and written informed consent for the percutaneous LAAC procedure was obtained from all patients.

### 2.2 TEE examination

The two‐dimensional (2D) and/or three‐dimensional (3D) TEE examination was performed 24 h preprocedurally using a PHILIPS EPIQ7C device (PHILIPS) in all patients. TEE was performed by two experienced echocardiographers, and the best images were recorded. The LAA size was assessed, and the presence of an LA thrombus was ascertained. During the 2D assessment, the LAA was visualized at 0°, 45°, 90°, and 135°, and measurements of maximum and minimum diameters of the LAA orifice were obtained from orthogonal planes (0° and 90°, and 45° and 135°), from the origin of the left circumflex artery to the roof of the LAA. For patients who underwent 3D TEE, images of the LAA were obtained using a single beat acquisition with a volume size large enough to include the entire LAA. The 3D datasets were digitally stored for offline analysis, and the maximum and minimum diameters of the LAA orifice were measured from the short‐axis view. The maximum diameter of the LAA was used as the reference to choose the device. The eccentricity index was calculated as the maximum/minimum diameter ratio to assess the LAA orifice geometry.

### 2.3 Percutaneous LAAC

All procedures were performed with the patient under uninterrupted anticoagulation. The device size was selected by the operator according to the preprocedural TEE measurements and intraprocedural LAA angiography results. The methods used for device implantation have been published previously. Briefly, the Watchman device was implanted through transseptal approaches using catheter‐based delivery systems. After a transseptal puncture, the transseptal sheath was exchanged with a delivery sheath, and intravenous heparin was administered to achieve an activated clotting time of more than 250 s. The device was advanced into the LAA through the delivery sheath and deployed via sheath retraction. TEE was performed to confirm the LAAC device position. A successful device position was defined as no or minimal contrast peridevice leakage (PDL) ≤ 5 mm into the LAA. A gentle tug test was performed to ensure device stability. The device was released after confirmation of an adequate position and a tug test.

Transthoracic echocardiography was performed to detect the presence of pericardial effusion on Postprocedural Day 1. Preprocedural adverse events, including stroke/TIA, major or minor bleeding events, and vascular complications (e.g., arteriovenous fistula, femoral hematoma, and pseudoaneurysm), were recorded for analysis.

### 2.4 Antithrombotic medication after LAAC

The antithrombotic medication strategy after LAAC was chosen by the operators based on the stroke and bleeding risk. For the first 45 days after the procedure, most patients have been treated with OACs (warfarin with an international normalized ratio of 2–3, 15–20 mg rivaroxaban once daily, or 110–150 mg dabigatran twice daily). If there was no PDL > 5 mm or a device‐related thrombus (DRT) assessed by TEE 45 days after LAAC, OAC was discontinued. Dual antiplatelet therapy (dual APT, 100 mg aspirin and 75 mg clopidogrel once daily) was initiated between 45 days and 6 months, and a single APT (100 mg aspirin or 75 mg clopidogrel once daily) was initiated after 6 months. If there was PDL > 5 mm or a DRT assessed by TEE at 45 days after LAAC, antithrombotic medication was continued until PDL > 5 mm and DRT disappeared. Computed tomography (CT) was the alternative if the patient refused to undergo TEE.

### 2.5 Clinical outcomes

The outcomes of the Watchman device determined by TEE follow‐up were collected, including PDL, DRT, and shoulder protrusion. The primary efficacy and safety events were recorded. The primary efficacy endpoint included ischemic stroke or systemic embolism. The primary safety endpoint included both procedure‐related events (e.g., pericardial effusion that required intervention, procedure‐related stroke, or device embolization) and major bleeding events during follow‐up. Major bleeding was defined according to the Bleeding Academic Research Consortium criteria (Type 3 or higher).^11^

### 2.6 Statistical analysis

Based on the preprocedural TEE measurements, the study patients were divided into two groups: patients with a small LAA orifice diameter (<17 mm; small LAA group) and patients with the indicated LAA orifice diameter (17–31 mm; indicated LAA group).

The rate of primary efficacy events in the PROTECT AF trial after 3.9 years of follow‐up was 8.4%.^12^ In our previous study, the incidence of primary efficacy events was 2.9% after 3.2 years of follow‐up.^13^ In the present study, we hypothesized that the long‐term safety and efficacy of LAAC were comparable between the small LAA group and indicated LAA group. For the noninferiority hypothesis, using a 10% noninferiority margin, using 80% power and 5% false‐positive (type I error) rate, assuming an efficacy of 91.6% for the small LAA group and 97.1% for indicated LAA group, using allocation ratio of 1:15, 21 and 316 patients were required for small LAA group and indicated LAA group, respectively.

Normally distributed continuous variables are expressed as the mean (standard deviation), while the median (interquartile range) is used for variables with a skewed distribution. Categorical variables are expressed as absolute numbers (percentages). Continuous variables were compared using the *t*‐test and Mann–Whitney *U* test for normally and nonnormally distributed data, respectively. Categorical variables were compared using the *χ* ^2^ test or Fisher's exact test where appropriate. All analyses were performed by SPSS 19.0 (IBM), and *p* < .05 was considered statistically significant.

## 3 RESULTS

### 3.1 Baseline characteristics

**Figure 1:**
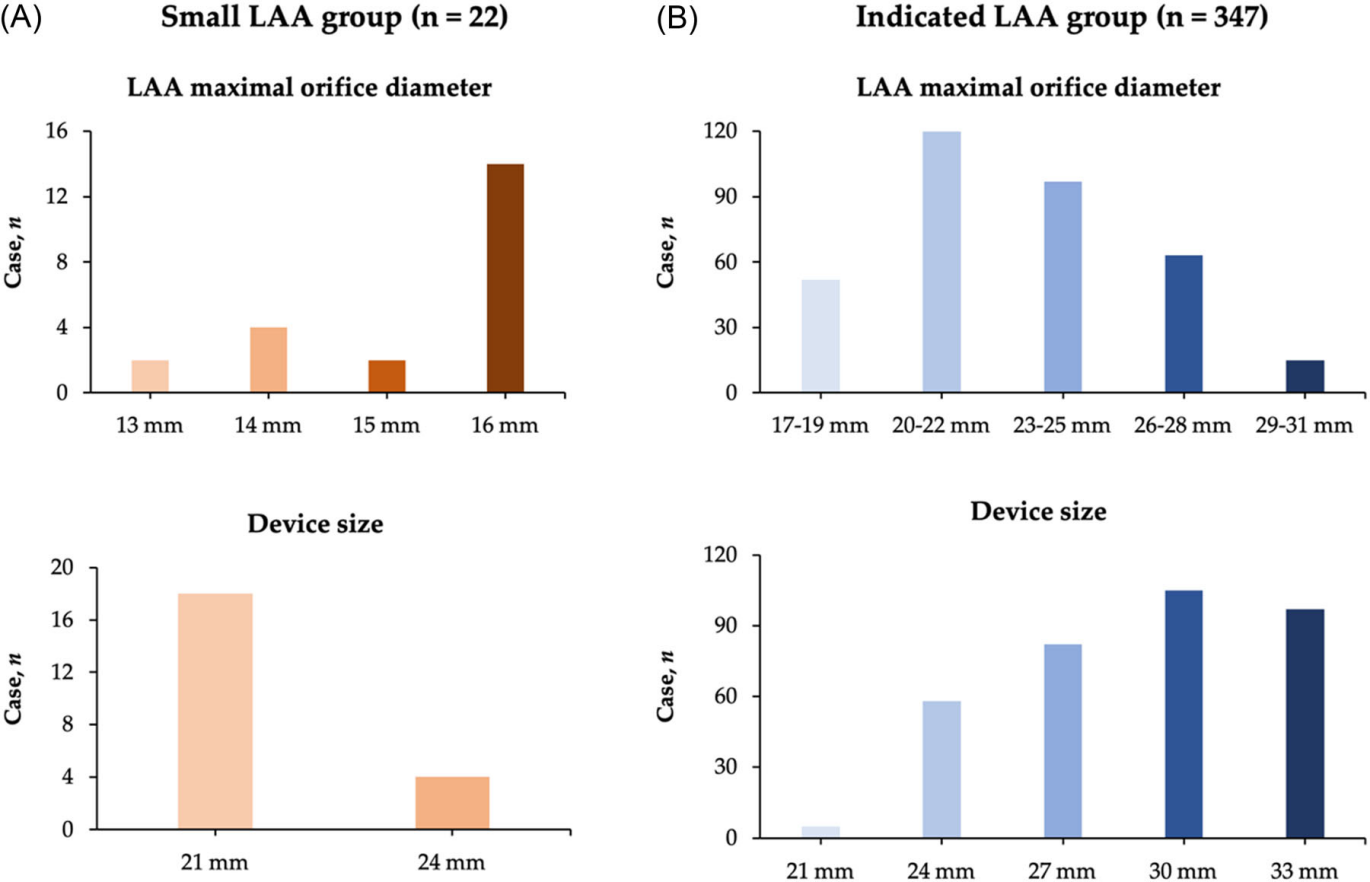
The distribution of the LAA maximal orifice diameter and implanted Watchman device diameter in the small LAA group (A) and indicated LAA group (B). LAA, left atrial appendage.

A total of 369 patients with AF under LAAC using the Watchman device were included in this study. All patients received a 2D TEE examination. 3D TEE was performed in 281 patients (76.2%). The study patients were divided into a small LAA group (*n* = 22) and an indicated LAA group (*n* = 347) (Table 1). The percentage of persistent AF was lower in the small LAA group (54.5% vs. 77.5%, *p* = .014). The patients in the indicated LAA group had a greater LA diameter (44.5 ± 6.6 mm vs. 39.2 ± 8.0 mm, *p* < .001), LAA maximal orifice diameter (23.0 ± 3.3 mm vs. 15.3 ± 1.1 mm, *p* < .001), and LAA minimal orifice diameter (18.8 ± 3.1 mm vs. 13.5 ± 2.1 mm, *p* < .001). The CHA_2_DS_2_‐VASc and HAS‐BLED scores were comparable between the two groups. The distribution of the LAA maximal orifice diameter is shown in Figure 1.

**Table 1:** Baseline characteristics

	Small LAA group	Indicated LAA group	*p* Value
*n*	22	347	
Age, years	70.9 ± 8.8	69.4 ± 8.6	.438
Male, *n* (%)	12 (54.5)	225 (64.8)	.329
Body mass index, kg/m^2^	23.5 ± 3.2	24.4 ± 3.4	.199
Persistent AF, *n* (%)	12 (54.5)	269 (77.5)	.014
Hypertension, *n* (%)	17 (77.3)	237 (68.3)	.378
Diabetes, *n* (%)	5 (22.7)	62 (17.9)	.057
Congestive heart failure, *n* (%)	2 (9.1)	38 (11.0)	1.000
Coronary heart disease, *n* (%)	2 (9.1)	18 (5.2)	.338
Previous TIA/stroke, *n* (%)	18 (81.8)	247 (71.2)	.282
Previous bleeding, *n* (%)	1 (4.5)	73 (21.0)	.094
CHA_2_DS_2‐_VASc score	5.0 ± 1.6	4.5 ± 1.5	.207
HAS‐BLED score	3.0 ± 1.2	3.0 ± 1.0	.842
LA diameter, mm	39.2 ± 8.0	44.5 ± 6.6	<.001
LVEF, %	65.1 ± 8.3	61.8 ± 7.0	.039
LAA maximal orifice diameter, mm	15.3 ± 1.1	23.0 ± 3.3	<.001
LAA minimal orifice diameter, mm	13.5 ± 2.1	18.8 ± 3.1	<.001
Eccentricity index of LAA orifice	1.18 ± 0.13	1.22 ± 0.15	.292

Abbreviations: AF, atrial fibrillation; LA, left atrium; LAA, left atrial appendage; LVEF, left ventricular ejection fraction; TIA, transient ischemic attack.

### 3.2 Periprocedural data

As shown in Table 2, the Watchman device was successfully implanted in all patients. The operation time and X‐ray exposure time were similar between the two groups. The mean device sizes were 21.5 ± 1.2 mm and 29.0 ± 3.3 mm in the two groups, respectively. LAA maximal orifice diameter by angiography was similar to that measured by preprocedural TEE. The distribution of implanted Watchman device types is displayed in Figure 1. For the small LAA group, a 21 mm device (18/22; 81.8%) was mostly used. Mean device compression was higher in the small LAA group (29.0 ± 3.3% vs. 15.1 ± 6.9%, *p* < .001). There were 48 patients (13.8%) with PDL < 5 mm in the indicated LAA group and 3 (13.6%) such patients in the small LAA group. Four patients (1.2%) in the indicated LAA group experienced pericardial effusion, and none experienced pericardial effusion in the small LAA group. No stroke/TIA occurred in either group.

**Table 2:** Periprocedural data

	Small LAA group	Indicated LAA group	*p* Value
*n*	22	347	
Operation time, min	58.0 (45.8, 84.5)	60.0 (50.0, 74.0)	.526
X‐ray exposure time, min	6.1 (4.3, 8.3)	6.4 (5.0, 8.5)	.344
LAA maximal orifice diameter by angiography, mm	16.1 ± 1.6	22.8 ± 3.2	<.001
Device size, mm	21.5 ± 1.2	29.0 ± 3.3	<.001
Release attempt, times	1.2 ± 0.4	1.3 ± 0.7	.479
Device compression, %	29.0 ± 3.3	15.1 ± 6.9	<.001
Peridevice leakage, *n* (%)			.839
>5 mm	0	0	
3–5 mm	0	8 (2.3)	
<3 mm	3 (13.6)	40 (11.5)	
None	19 (86.4)	299 (86.2)	
Complication, *n* (%)			
Stroke/TIA	0	0	1.000
Device embolization	0	0	1.000
Pericardial effusion	0	4 (1.2)	1.000
Vascular complications	0	5 (1.4)	1.000

Abbreviations: LAA, left atrial appendage; TIA, transient ischemic attack.

### 3.3 Follow‐up results

Follow‐up results are displayed in Table 3. OAC was prescribed in most patients within 45 days after LAAC (100% in the small LAA group and 96.5% in the indicated LAA group). DRT was detected in one (4.5%) patient in the small LAA group and five (1.4%) in the indicated LAA group (*p* = .310). Four patients were treated with dabigatran, and the other two patients were treated with rivaroxaban at discharge. After prolonging the duration of OAC medication, the thrombi disappeared, as confirmed by repeated TEE. No PDL > 5 mm was detected in either group on repeated TEE. PDL < 5 mm was found in 3 (13.6%) and 48 (13.8%) patients in the small LAA group and indicated LAA group, respectively. The percentage of shoulder protrusion was higher in the small LAA group, but the difference was statistically insignificant (18.2% vs. 8.9%, *p* = .144).

**Table 3:** Follow‐up results

	Small LAA group	Indicated LAA group	*p* Value
*n*	22	347	
Follow‐up time, years	4.2 ± 1.5	4.1 ± 1.6	.956
The primary efficacy endpoint, *n* (%)			
Ischemic stroke	1 (4.5)	4 (1.2)	.266
Systemic embolism	0	0	1.000
The primary safety endpoint, *n* (%)			
Procedure‐related events^a^	0	4 (1.2)	1.000
Major bleeding events	0	0	1.000
TEE follow‐up, *n* (%)			
DRT	1 (4.5)	5 (1.4)	.310
Peridevice leakage			.623
>5 mm	0	0	
3–5 mm	1 (4.5)	9 (2.6)	
<3 mm	2 (9.1)	39 (11.2)	
None	19 (86.4)	299 (86.2)	
Shoulder protrusion	4 (18.2)	31 (8.9)	.144
Antithrombotic medication, *n* (%)			.566
Warfarin	3 (13.6)	63 (18.2)	
Dabigatran	14 (63.6)	152 (43.8)	
Rivaroxaban	5 (22.7)	120 (34.6)	
Dual APT	0	8 (2.3)	
Single APT	0	4 (1.2)	

Abbreviations: APT, antiplatelet therapy; DRT, device‐related thrombus; LAA, left atrial appendage; TEE, transesophageal echocardiography.

^a^ See details in Table 2.

**Figure 2:**
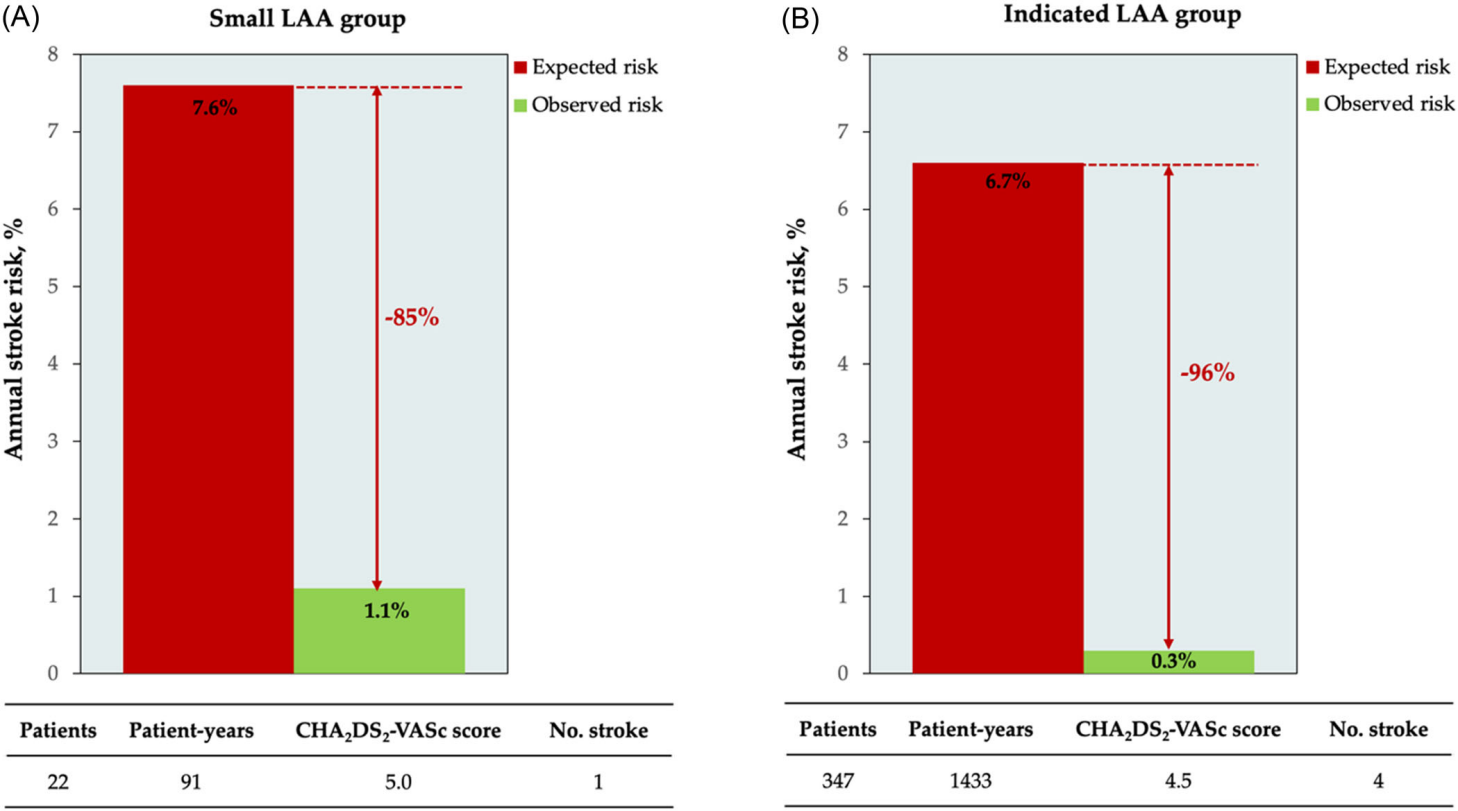
Effectiveness of LAA closure using the Watchman device in the reduction of stroke risk based on annual risk predicted by the CHA_2_DS_2_‐VASc score in the small LAA group (A) and indicated LAA group (B). LAA, left atrial appendage.

After a mean follow‐up period of 4.1 ± 1.6 years, one patient in the small LAA group (4.5%) and four in the indicated LAA group (1.2%) suffered primary efficacy endpoint (all ischemic stroke) (*p* = .266). The annual ischemic stroke rate was 1.1/100 person‐years (85% reduction compared to expected risk) and 0.3/100 person‐years (96% reduction compared to the expected risk) for the small LAA group and indicated LAA group, respectively (Figure 2). All patients recovered without sequelae after conventional treatment. There was no primary safety end point during follow‐up.

## 4 DISCUSSION

### 4.1 Main findings

This study first compared the long‐term safety and efficacy of LAAC with Watchman between patients with a small LAA orifice (<17 mm) and those with the indicated LAA orifice diameter (17–31 mm). The results showed that the Watchman device could also be successfully implanted in patients with a small LAA orifice measured by TEE. The periprocedural complications and long‐term clinical outcomes during follow‐up were comparable between patients with small and indicated LAA orifice diameters.

### 4.2 LAAC in small LAA: Potential risk and feasibility

LAAC with the Watchman device is indicated to reduce the risk of TE in patients with AF.^14^, ^15^ The Boston Scientific Instructions for Use for the Watchman recommends a maximal LAA orifice diameter between 17 and 31 mm to accommodate current device sizes.^5^ There are several potential risks of LAAC in patients with an LAA orifice diameter <17 mm due to an excessive compression ratio. A larger occluder may result in a significant shoulder on either the mitral or limbus side and may embolize into the LA during deployment. In addition, an excessive compression ratio may damage the LAA wall and lead to major bleeding events. However, LAAC with the Watchman device in patients with a small LAA orifice measured by TEE may be feasible for the following reasons.

First, there are some factors that may affect the measurement of LAA size by TEE. Usually, TEE examination was performed from four different planes (0°, 45°, 90°, and 135°) to measure the LAA size. However, the maximal LAA orifice diameter may be found on other degree planes. Preprocedural TEE involves at least 6 h of fasting. This may affect the volume status of the patient, which in turn may affect the LAA size. Some studies have shown that volume loading increases LAA dimensions by 2 mm and more accurately predicts the final device size.^16^, ^17^ Osmancik et al.^18^ and Saw et al.^19^ both found that CT measurement of the LAA size was more accurate and suitable for the choice of occluder than TEE. Therefore, CT may be an optional choice because most patients are not required to restrict fluids before CT.

Second, the LAA is a very flexible structure. A recent investigation showed that the diameter of the LAA increases by 2 mm after implantation with the Watchman device and the LAA orifice changes from an oval to a more circular shape.^20^ Another study found that the compression rate was smaller during follow‐up than during the procedure.^21^ The above studies suggested that the LAA adapts to the shape of the occluder after implantation.

Third, more device choices may favor the feasibility of LAAC in patients with a small LAA. The new‐generation Watchman FLX device is available in 20, 24, 27, 31, and 35 mm sizes and the recommended range of suitable implant LAA orifice diameter is 14–31.5 mm.^22^ A study showed that the application of Watchman FLX in patients with a failed Watchman attempt (*n* = 11) or off‐label LAA anatomy (*n* = 88; 51 with LAA orifice <17 mm) was safe and efficient.^23^ However, whether Watchman FLX can be used in patients with an LAA orifice <14 mm is unknown. In addition, Watchman FLX is now unavailable in some regions.

### 4.3 Safety and efficacy of LAAC in patients with small LAA

The recommended range of compression rate is 8%–20%.^5^ In the present study, a total of 22 patients with LAA < 17 mm measured by TEE were successfully implanted with the Watchman device. The range of maximal LAA orifice diameter in the small LAA group was 13–17 mm, with an average compression ratio of 29% and a maximal compression ratio of 37%. In the study of Venkataraman et al.,^9^ the Watchman device was successfully implanted into 32 patients with a maximal LAA orifice diameter <17 mm (14–16 mm), with an average compression ratio of 24% and a maximal compression ratio of 43%. No pericardial effusion, device embolization, or stroke/TIA occurred periprocedurally in the study of Venkataraman et al^9^ and the present investigation. In the EWOLUTION study, the device oversizing percentage was over 30% in nearly 1/3 patients, indicating that overcompression rate of over 20% was not uncommon in clinical practice. However, multivariable Cox regression analysis showed no significant association between device oversizing and pericardial effusion.^8^ Therefore, Watchman device implantation in patients with a small LAA orifice, which results in a relatively greater compression rate than the recommended range (8%–20%), may be safe and feasible.

PDL is common after Watchman device implantation. A wide range of PDL frequencies has been reported across previous studies. The PROTECT‐AF study showed that Watchman had a 32% incidence of PDL at 12 months.^24^ PDL was detected in 32% of patients by TEE after Amplatzer device implantation.^25^ In the study of Venkataraman et al, TEE after LAAC in a small LAA demonstrated a 1–4 mm PDL in 6% of patients.^9^ In the present study, the incidence of PDL detected by follow‐up TEE was 13.6% in the small LAA group, which was comparable with that in the indicated LAA group as well as in previous studies. DRT is one of the major concerns after LAAC. In the EWOLUTION registry, the diagnosis of DRT was made in 4.1% of patients who underwent LAAC and completed at least one TEE follow‐up.^26^ In addition, the incidence of DRT in the PROTECT‐AF study was 4.2%.^27^ In the current investigation, the incidence of DRT (4.5%) in the small LAA group was similar to that in previous studies.

Data from PREVAIL and PROTECT‐AF trials showed that the incidence of stroke was 1.68 and 1.36/100 patient‐years after 5 years of follow‐up, respectively.^28^ In the EWOLUTION registry, the annual stroke risk was 1.3/100 patient‐years.^29^ The only published study concerning LAAC in patients with a small LAA only had short‐term follow‐up results.^9^ In our study, the annual stroke risk in the small LAA group was slightly higher than that in the indicated LAA group (1.1/100 person‐years vs. 0.3/100 person‐years) but comparable to prior investigations. Therefore, LAAC with the Watchman device for stroke prevention in patients with a small LAA orifice may be efficient.

### 4.4 Limitations

There are several limitations of our study. First, the major limitation is that it was a single‐center, observational, and retrospective study with a small sample size, which may limit the interpretability. However, to the best of our knowledge, this is the first investigation to report the long‐term outcomes of patients with a small LAA who underwent LAAC. Second, only the Watchman device was used in the present study. Whether other types of occluders are suitable for LAAC in small LAAs is still unknown. Third, the smallest maximal LAA orifice diameter included in this study was 13 mm. LAAC with the Watchman device in patients with a maximal LAA orifice diameter <13 mm may yield different results. Finally, alternative imaging examinations, such as cardiac CT, may have provided a more accurate assessment of maximal LAA orifice diameter. However, data were not available in this analysis.

## 5 CONCLUSION

The safety and efficacy of LAAC with the Watchman device were comparable between patients with small (<17 mm) and indicated (17–31 mm) LAA orifice diameters. Therefore, patients with a maximal LAA orifice diameter <17 mm measured by TEE may not be considered to have a prohibitive LAA anatomy, and satisfactory LAAC may also be achieved with the Watchman device in such patients.

## CONFLICT OF INTEREST

The authors declare no conflict of interest.

## Data Availability

The data supporting this study's findings are available from the corresponding author upon reasonable request.
